# Data for assessment of soil water extractable and percolation water dissolved organic carbon in watersheds

**DOI:** 10.1016/j.dib.2019.104779

**Published:** 2019-11-06

**Authors:** Fabien Rizinjirabake, David E. Tenenbaum, Petter Pilesjö

**Affiliations:** a Lund University, Department of Physical Geography and Ecosystem Science, Sweden; b University of Rwanda, College of Science and Technology, Biology Department, Rwanda; c Centre for Middle Eastern Studies, Lund University, Sweden

**Keywords:** Water extractable dissolved organic carbon, Percolation water dissolved organic carbon, Antecedent precipitation indices, Mean antecedent temperature, Topographic position index, Curvature, Soil properties

## Abstract

(“Sources of soil dissolved organic carbon in a mixed agricultural and forested watershed in Rwanda”, [6])

This data article presents water extractable organic carbon (WEOC), percolation water dissolved organic carbon (pDOC), and mean antecedent precipitation indices (API) and mean antecedent temperature (MAT) data. The article also presents edaphic properties such soil texture elements, total organic carbon (TOC), total nitrogen (TN), cation exchange capacity (CEC), iron (Fe), and aluminum (Al). Additionally, the article presents topography attributes such including topographic position index (TPI) and curvature. All these data were used to analyze both WEOC and pDOC dynamics in the Rukarara River Watershed (RRW), Rwanda. WEOC and soil properties data were analyzed from sampled 52 soil composites samples collected during from October to December 2016 using 53 × 50 mm rings. Data of pDOC were analyzed from percolation water samples collected using a zero tension lysimeters on various dates during the period from Jun 2015 to Jun 2017. API and MAT data for various antecedent days were calculated on basis of rainfall and air temperature data recorded at three stations within the RRW using respectively tipping bucket rain gauges and sensors installed at three sites located representing the main land use land cover classes within the RRW.

**Table:** Specifications Table

Subject area	Land management
More specific subject area	Land management deals with terrestrial ecosystems and is used for organic agriculture, reforestation, and water resource management. Managing land resources sustainably is important to ensuring important ecosystem services such as watershed protection, biodiversity conservation and carbon sequestration.
Type of data	Tables
How data was acquired	Topsoil and percolation water samples were collected and transported to laboratory for analysis. Samples were collected by 53 × 50 mm rings and zero tension lysimeters respectively for soil and percolation water. Sol samples were collected within four main land cover classes and percolation water samples in pits at the foot of hillslopes.
Data format	Raw and Analyzed
Experimental factors	We added 1ml of sulfuric acid (H_2_SO_4_) at each percolation water sample to inhibit microbial activity. Soil and water percolation samples were transported to the laboratory on ice to reduce eventual microbial activity. Soil samples were dried, as soon as possible, in an oven at 105 °C for 48 hours to avoid microbial degradation of organic matter in samples.
Experimental features	Dried soil samples were soaked with distilled water for 48 hours and the supernatant solution used to analyze the amount of water extractable organic carbon (WEOC). WEOC and pDOC data were analyzed on TOC analyzer.
Data source location	Rukarara River watershed (29⁰15–29⁰35E and 2⁰20N-2⁰35S)
Data accessibility	Data is with this article
Related research article	[6]

**Table:** 

**Value of the Data** • Provided WEOC and pDOC data are useful for land and water resource management. Farmers, foresters, environmentalists, decision makers and researchers can benefit from these data; for example, researchers can use these data to quantify effect of climate change on carbon dynamics and decision makers to set up strategies for watershed protection and/or restoration. • Soil properties data are also useful for land and water resource management. Farmers, environmentalists, and decision makers can use these data to study soil and inland water degradation via WEOC and pDOC export and thus on the potential effects of climate land cover change on terrestrial and aquatic services. Soil properties can also be used to reveal the responses of WEOC and/or pDOC to soil properties and therefore to carbon dynamics within watersheds. • Antecedent precipitation and temperature data are useful for environment management under ongoing global change. These data can be used by the scientific community and environmental managers to determine the role of these antecedent climate conditions to reduce or exacerbate effects of climate change on ecosystems health. API and MAT represent not only the antecedent wetness and temperature, but also the effects of the current precipitation and temperature. Thus, API and MAT are interesting measures of climate variability. • Slope position and curvature data are useful in determining flat, concave and convex landscape areas and can be used to separate effects of land use land cover/land management on WEOC and pDOC. This information is important, for example, for farmers and land managers.

## 1 Data

This data article presents data of WEOC, pDOC, and soil properties including total organic carbon (TOC), total nitrogen (TN), cation exchange capacity (CEC), iron (Fe), aluminum (Al), and soil texture. Additionally, the article presents pDOC, antecedent precipitation indices (API), mean antecedent temperatures (MAT), slope position, and curvature data for a tropical watershed in the Rukarara River Watershed (RRW) in Rwanda. All data in this article are presented in the form of tables. For each table, standard deviation and range were given to provide information upon the variation of presented data. WEOC data and LULC types were presented for each sampling plot. Regarding pDOC data, they were presented for each sampling date. Alongside different values of soil properties per plots, we presented their corresponding normalized values and LULC types. The table for API data presents API values calculated at different dates for 56, 42, 28, 21, 17, 14, 7, and 5 antecedent days at natural forest, tea and farm sites. MAT data were also presented for different dates and three sites but for 100, 56, 42, 28, 21, 14, and 7 antecedent days. Slope position data were presented per plot and we gave LULC types, slopes, topographic position index (TPI), and WEOC. Curvature data were presented also per plot and we indicated corresponding curvature values, LULC types, and WEOC.

## 2 Experimental design, materials, and methods

### 2.1 WEOC data

Soil samples were collected in top 20 cm of the soil at locations in natural forest, tree plantations, tea plantations and croplands within the RRW. Samples were collected during the period October–November 2016, using soil sample rings (53 × 50 mm) at 52 sampling plots (2  m × 2  m). Plots were selected on basis of the main LULC classes. In each plot, soil samples were collected at 5 points configured in an X shape and spaced 2 m apart. All point soil samples were clearly labelled by indicating sampling date, plot code, and geographical coordinates of the plot, and were transported to a laboratory for analysis of WEOC. In total 260 soil samples were collected and used to obtain 52 soil composite samples. From the samples, we removed roots and stones before forming composite soil samples. The following Table 1 presents WEOC data for the 52 sampling plots selected in the four main LULC classes. Precision measures including range and standard deviation.

Data Table 1: Topsoil WEOC data in the main LULC classes within the RRW (See attached MS Excel file).

### 2.2 Soil properties data

Soil samples were used not only to analyze WEOC but also soil properties including soil texture, TOC, TN, CEC, Fe, and Al. Amounts of WEOC were analyzed using a the Zhang et al. modified method [9]: soil samples dried in an oven at 105 °C for 48 hours (0.30 Kg) were soaked each in 75 ml of distilled water for 48 hours and resulting supernatant solution was used to analyze WEOC (mgC/L) in a TOC analyzer. The supernatant solution was before filtered using a 0.45 μm nylon filter before it was analyzed.

Sand, silt and clay contents (%) were analyzed using the improved Bouyoucos method [1]; the TOC (%) by the Loss On Ignition (LOI) method [3]; the TN (%) by the micro-Kjeldahl digestion method [2], the CEC (mEq/100g) by the Sodium acetate method [7], and the Fe and Al (ppm) by the Sodium Tetraborate method, followed by the atomic absorption spectroscopy (AAS) method [5]. Precision measures including range and standard deviation.

Data Table 2: Raw and normalized soil composite topsoil samples within the RRW (See attached MS Excel file).

### 2.3 pDOC data

Samples to analyze percolation water DOC were collected at various dates from the period Jun 2015 to Jun 2017. Samples were collected in pits at the foot of hillslopes in plastic sampling bottles using zero tension lysimeters. Samples were transported on ice to the University of Rwanda (UR) laboratory complex for pDOC analysis on a TOC analyzer. Samples were filtered using 0.45 μm nylon filters before analysis on TOC analyzer. Precision measures including range and standard deviation.

Data Table 3: Percolation water organic carbon at foot of hillslopes within the RRW (See attached MS Excel file).

### 2.4 API and MAT data

API and MAT data to analyze the relationship between percolation water DOC and climate variability, data were calculated based on temperature and rainfall data. These temperature and rainfall data were recorded from June 2015 to June 2017 using respectively sensors (PT2X and minidivers) and tipping bucket rain gauges, with integrated data loggers (OMC-210-2). Sensors and rain gauges installed in natural forest, tea plantations and croplands sites. Tables 5 and 7 present, respectively, raw temperature and rainfall data at the three sites within the RRW, whereas Tables 4 and 6 present respectively MAT and API data recorded at three sites. Precision measures including standard deviation and range.

Data Tables 4 and 5: Raw temperature and MAT data within the RRW (See attached MS Excel file): Raw rainfall and API data within the RRW (See attached MS Excel file).

### 2.5 Topography attributes data

Topographic position index (TPI) values of the studied watershed were calculated in the ArcMap 10.2.2 environment using Topography tools 10.1 [4] from a 10 m DEM. TPI values were used to determine slope positions (ridge, upper slope, middle slope, flat slope, lower slopes and valleys) using the Weiss method [8]. Regarding curvature, its values were calculated in ArcMap 10.5.1 environment from the 10 m DEM under Spatial Analyst Tools. Negative, null, and positive values of the curvature correspond respectively to concave up, flat and convex areas. Tables 8 and 9 present data of slope position and curvature within the RRW.

Data Tables 8 and 9 : Slope position and curvature data within the study area (See attached MS Excel file).
